# Effective control measures considering spatial heterogeneity to mitigate the 2016–2017 avian influenza epidemic in the Republic of Korea

**DOI:** 10.1371/journal.pone.0218202

**Published:** 2019-06-13

**Authors:** Jonggul Lee, Youngsuk Ko, Eunok Jung

**Affiliations:** 1 National Institute for Mathematical Sciences, Daejeon, Republic of Korea; 2 Mathematic department, Konkuk University, Seoul, Republic of Korea; Montana State University, UNITED STATES

## Abstract

During the winter of 2016-2017, an epidemic of highly pathogenic avian influenza (HPAI) led to high mortality in poultry and put a serious burden on the poultry industry of the Republic of Korea. Effective control measures considering spatial heterogeneity to mitigate the HPAI epidemic is still a challenging issue. Here we develop a spatial-temporal compartmental model that incorporates the culling rate as a function of the reported farms and farm density in each town. The epidemiological and geographical data of two species, chickens and ducks, from the farms in the sixteen towns in Eumseong-gun and Jincheon-gun are used to find the best-fitted parameters of the metapopulation model. The best culling radius to maximize the final size of the susceptible farms and minimize the total number of culled farms is calculated from the model. The local reproductive number using the next generation method is calculated as an indicator of virus transmission in a given area. Simulation results indicate that this parameter is strongly influenced not only by epidemiological factors such as transmissibility and/or susceptibility of poultry species but also by geographical and demographical factors such as the distribution of poultry farms (or density) and connectivity (or distance) between farms. Based on this result, we suggest the best culling radius with respect to the local reproductive number in a targeted area.

## Introduction

During the 2016-2017 winter season, the epidemic of highly pathogenic avian influenza (HPAI) in the Republic of Korea led to high mortality rates in domestic poultry and put a serious economic burden on the poultry industry. By April 4, 2017, 383 farms were reported to be infected by the HPAI virus (subtype H5N6 and H5N8), and approximately 3.7 million poultry (3154 thousand chickens and 332 thousand ducks) from 946 farms were culled (i.e., depopulated) [1]. As most of the culled chickens are layer chickens (2518 thousand), it disrupted the egg supply and led to a surge in the egg price [2, 3].

Avian influenza (AI) virus is usually not serious in wild birds, but it causes a critical infection in domestic poultry such as chickens and ducks. By ability of the virus to cause disease and mortality in chickens, the infections divided into two forms [4]: low pathogenic avian influenza (LPAI), causing mild symptoms such as decreasing egg production, and the HPAI, causing infected hosts to have high mortality rates up to 90-100% within 2 days [5]. AI is spread by direct contact with birds. The AI virus is mainly transmitted through the feces or respiratory tract of infected birds [6]. Poultry can be infected with the AI virus via between-farm movements of vehicles, people, feed and poultry [2, 7].

Culling is the process of killing poultry, both infected and uninfected, in areas around the infected region to rapidly contain the spread of an infectious disease with the aim of finally eradicating the disease [8]. Based on a zoning strategy, in practice, authorities mainly carry out following two culling strategies.

- Infected premises (IP) culling: killing all poultry in a farm or small area where the sick or dead poultry are diagnosed as AI-positive.
- Preemptive (PE) culling: preemptively killing all poultry within a protect area, which is declared a area around the IP up to 3 *km* in the Republic of Korea.

Although such stamping-out policies have been considered to be one of the main control measures for decades, there remain some controversies about animal welfare and effectiveness. Especially, when PE culling is conducted in dense area of farms, it inevitably leads to a huge number of killed poultry. Over 10 million chickens were culled in the Republic of Korea to mitigate H5N1 in 2008 [1]. The decreased poultry industry revenue in China from February to April 2004 caused by HPAI was estimated to 49.6% [9]. Nonetheless, a culling policy is widely adopted because of a dramatic reduction in the risk of dispersion in a short time by decreasing the infectious period of infected farms and also removing susceptible farms which can be infectious in advance.

Mathematical modeling has been used to understand the dynamics of AI epidemics and to provide insights on control measures against the spread of the AI virus. Compartmental SIR-type models without spatial heterogeneity were studied for human infection of the AI virus [10–12] with optimal control theory [13], AI transmission between wild birds and poultry [14], and impact of culling strategies on H5N1 infection in domestic bird populations [15, 16]. Many studies have explored the role of spatial heterogeneity and control measures in AI epidemics [17–23]. Agent-based models are beneficial to show warnings on mass culling strategy [17, 18]. Distance-dependent transmission probability between poultry farms provides transmission risk in a geographical region [19, 24]. Stochastic farm-to-farm transmission models have been used to investigate control measures dependent on spatial heterogeneity, including poultry farm density [19, 20]. The transmission characteristics of the HPAI virus were quantified by the estimation of the reproductive number and used it to evaluate the effectiveness of various control measures against HPAI epidemics in recent studies [19, 21–23, 25].

Even though many mathematical models have focused on various control measures against AI virus, there has been little research on various culling strategies considering the breeding type of poultry farms and the farm density in targeted areas. Moreover, it is still a challenge to suggest an effective control scenario based on the geographical distribution of poultry farms. In this study, we focus on the effects of various culling scenarios on the spread of avian influenza between domestic poultry farms in the Republic of Korea, taking into consideration their breeding types and geographical distributions. We introduce culling functions incorporating the farm density of targeted areas and describe the farm-based metapopulation model with these culling functions. The model is parameterized with AI data reported to the Ministry of Agriculture, Food and Rural Affairs (MAFRA) [1] for the 2016-2017 period. We introduce key parameters to quantify the local transmissibility and use it to compare culling scenarios to maximize the number of surviving farms after the AI epidemic.

## Materials and methods

### Epidemiological and geographical data

In this study, we focus on the 2016-2017 HPAI outbreak in the neighboring sixteen towns which are lower-level administrative divisions of Eumseong-gun and Jincheon-gun in Chungcheongbuk-do. Before the HPAI outbreak, there were 589 poultry farms (454 for chicken and 135 for duck) in these sixteen towns [26]. With the onset of the HPAI outbreak, starting from the town Maengdong, which is located at the center of Eumseong-gun and Jincheon-gun, 71 farms (14 for chicken and 57 for duck) were reported as infected and approximately 2 millions poultry were culled [1] in these sixteen towns.

**Fig 1:**
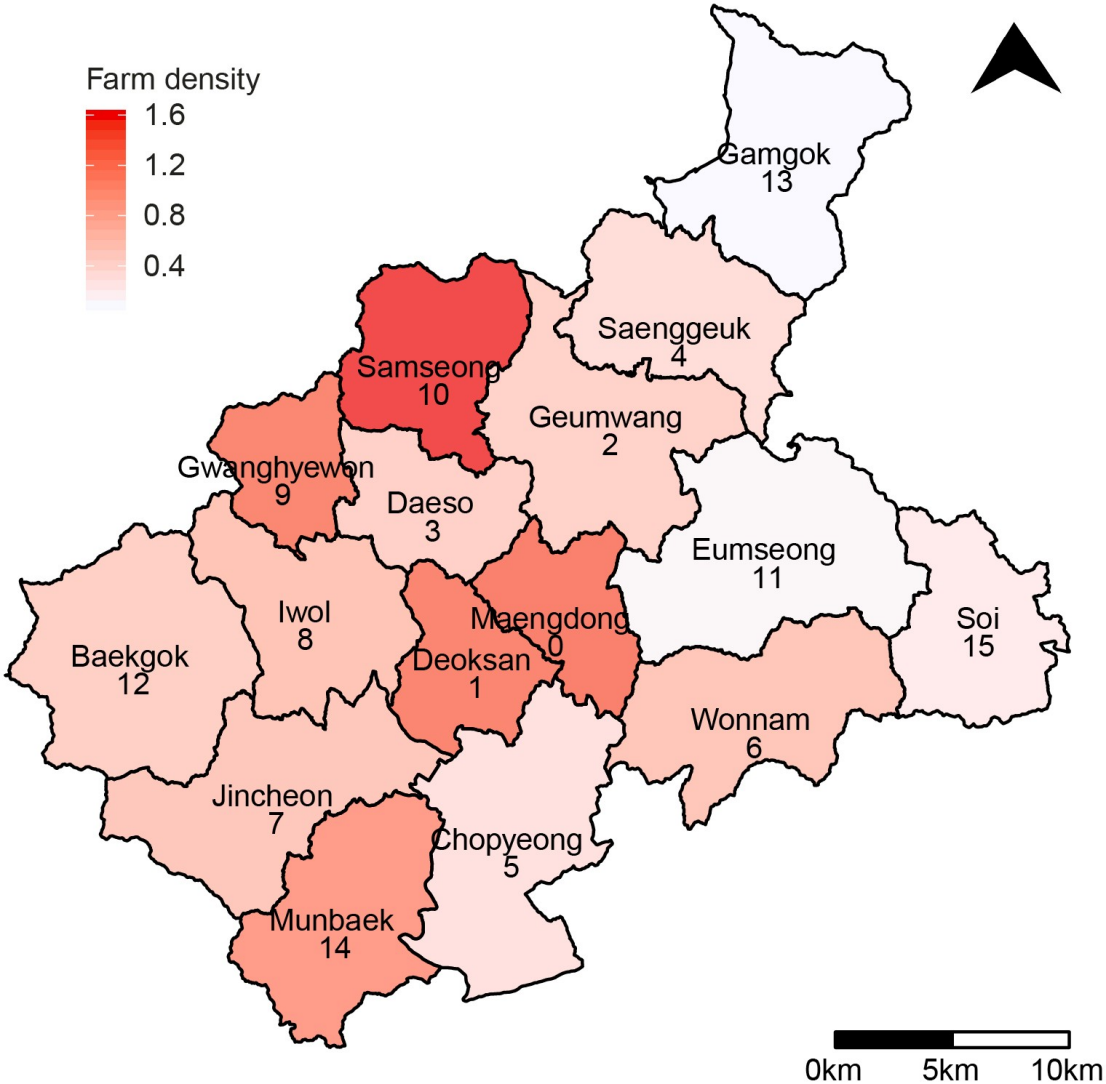
Map of Eumseong-gun and Jincheon-gun, which are the municipal-level divisions located in the central part of the Republic of Korea, including the density of poultry farms with white indicating low density and red indicating high density. The index of the town is written below its name. There are 16 towns; 9 towns in Eumseong-gun and 7 in Jincheon-gun. Reprinted from [27] under a CC BY license, with permission from National Geographic Information Institute, original copyright 2017.

The data in Table 1 shows the geographical and epidemiological information of the sixteen towns, and is used in our mathematical model. The sequential outbreak data for each type of poultry farms (S1 and S2 Tables) is used for estimation of model parameters. Note that each town is assigned an index in the ascending order of the distance from Maengdong, which is set to index 0. Matrix of pairwise distances between towns (S3 Table) is used in a transmission kernel of our model. Assuming that the farms are uniformly distributed in each town, the farm density is the number of farms per area. The distribution of farm density in a map is shown in Fig 1. The reported numbers of outbreak farms were 57 for duck and 14 for chicken. Maengdong had the largest reported number, i.e., 23, and Iwol-myeon had the second largest number, i.e., 11. The ratio of reported number of farms to total number of farms was 0.12. The ratio of reported duck farms to total number of duck farms was 0.42 while the ratio of reported chicken farms to total number of chicken farms was 0.03. These imply that the 2016-2017 HPAI outbreak occurred mostly at duck farms.

**Table 1:** Numbers of poultry farms and reported cases of sixteen towns in 2016-2017.

	Poultry farms			Outbreaks			
Town (Index)	Chicken	Duck	Total	Chicken	Duck	Total	Area (*km*^2^)
Maengdong (0)	14	33	47	1	22	23	34.69
Deoksan (1)	35	11	46	2	5	7	35.02
Geumwang (2)	29	9	38	0	4	4	71.33
Daeso (3)	6	15	21	0	3	3	38.17
Saenggeuk (4)	23	0	23	2	0	2	56.04
Chopyeong (5)	21	6	27	0	2	2	76.29
Wonnam (6)	38	5	43	1	1	2	64.77
Jincheon (7)	38	8	46	1	1	2	70.49
Iwol (8)	17	21	38	1	10	11	55.20
Gwanghyewon (9)	37	2	39	0	0	0	29.80
Samseong (10)	65	16	81	6	3	9	50.59
Eumseong (11)	11	0	11	0	0	0	86.42
Baekgog (12)	45	0	45	0	0	0	80.09
Gamgok (13)	4	2	6	0	2	2	69.48
Munbaek (14)	60	6	66	0	4	4	60.18
Soi (15)	11	1	12	0	0	0	48.90
Total	454	135	589	14	57	71	927.46

### Culling rate functions

In this subsection, we describe nonlinear culling rates as a function of the number of AI-confirmed farms. Constant culling rate is used for simplicity in compartmental models [14, 16], but it might be inappropriate to consider culling capacity or resource limitation [15, 22]. In practice, when a poultry farm is diagnosed positive for HPAI on either clinical diagnosis or laboratory analysis, PE culling is conducted on farms within a protect area which is a declared area around the reported farms of perimeter 3 *km* in general. The PE culling process is analogous to transmission of diseases; once an infected farm is identified and confirmed AI infection by a epidemiological surveillance, most farms having close contacts would be designated as dangerous contacts and depopulated soon. Therefore, it is reasonable to adopt nonlinear culling terms (or constant culling rates) [15] similar to nonlinear transmission terms such as density-dependent transmission [28, 29]. In this work, we employ the nonlinear culling terms in depopulation of the poultry farms. Under the limited culling strategy, we set the culling rate as a rational function of the number of reported farms. Two nonlinear culling types are considered in this study: PE culling and IP culling.

#### PE culling rate function

We set the PE culling rate as a rational function of the number of reported farms [15]. For culling by a reported farm (*R*), the number of susceptible farm (*S*) to be culled is proportional to density of those farms within a culling area of radius, *r*_*c*_, i.e., $(S/a)\pi r_{c}^{2}=(\pi r_{c}^{2}/a)S=DS$, where *D* is the fraction of culling area, $D=\pi r_{c}^{2}/a$ and *a* is the area. Then, rate of culling with a saturation factor is given as $\eta D\frac{SR}{A+R}$, where *η* and *A* are PE culling constant and decay constant for the PE culling rate, respectively. In this study, we define the nonlinear PE culling rate function as $\psi \left(R\right)=\eta D\frac{R}{A+R}$. Note that *ψ*(0) = 0, which means PE culling strategy is employed after an AI outbreak occurs.

#### IP culling rate function

**Fig 2:**
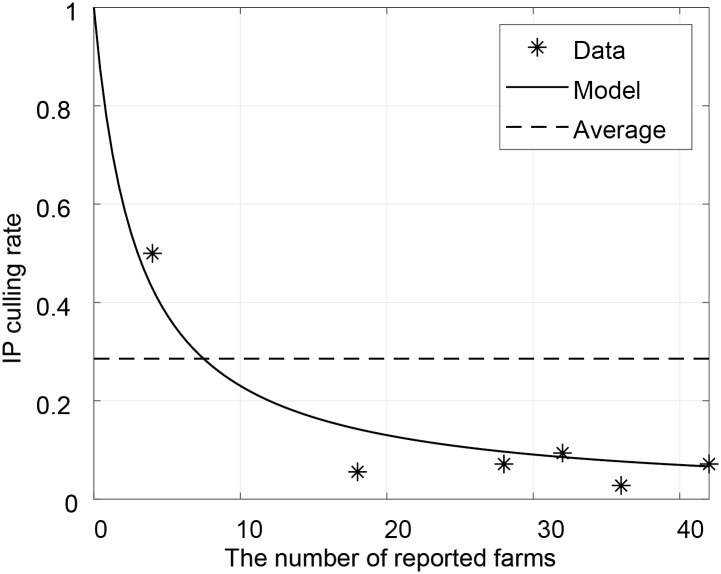
Estimation of IP culling rate. The stars and the solid curve denote the approximated IP culling rate with respect to the number of AI-reported farms and the corresponding calibrated curve, respectively. The dashed line represents constant culling rate averaged over data. The data for IP culling rate approximation is presented in S4 Table.

The IP culling process is a control strategy that removes the reported farms to stop disease spreading. Although emergency action guidelines for AI describe that culling procedure should be proceeded in a day [30], it is likely to take 2 days or more because of the simultaneously surging reported cases and limitation of the resources for control measures. We assume that the IP culling rate is a decreasing function of *R*, and given by $\phi \left(R\right)=\frac{\gamma B}{B+R}$, where *γ* is maximum culling rate and *B* is a decay constant. When *γ* is fixed as 1 [30], parameter *B* can be estimated from the IP culling rates with respect to the number of reported farms by the following approximation: ignoring the type of farm and patch, let *N*_*IP*_ be the number of IP culling obtained by data (S4 Table), i.e., *N*_*IP*_ = *ϕ*(*R*)*R*, then *ϕ*(*R*) = *N*_*IP*_/*R*. By integrating *dR*/*dt* from (2), we have $R=\int_{0}^{t}\left[I-\phi \left(R\right)R\right]=\int_{0}^{t}\alpha I-\int_{0}^{t}\phi \left(R\right)R$, where the left term is the cumulative number of AI-reported farms and the right term is the cumulative number of IP culling. These data are provided in S4 Table. In Fig 2, the stars and the solid curve denote the approximated IP culling rate with respect to the number of AI-reported farms and the corresponding calibrated curve, respectively. Fig 2 shows that the IP culling rate decreases as the number of reported farms increases. Note that the average of IP culling rate from the data might be overestimated when there are a large number of AI-reported farms.

### Metapopulation model with two species

Now we consider a compartmental model to incorporate spatial effects. Let *S*_*i*_, *I*_*i*_, *R*_*i*_ denote respectively the number of susceptible, infective but not identified, and reported farms in patch *i* for *i* = 0, …, 15. Note that in our model *I*_*i*_ and *R*_*i*_ are infectious, but *R*_*i*_ has less transmissibility because of control measures after the case is identified, such as isolation and restriction. Ignoring the species, the spatial-temporal model can be written for *i* = 0, …, 15 as follows

$$\begin{array}{c} \begin{array}{cc} \frac{dS_{i}}{dt} & =-\sum_{j=0}^{15}\beta (I_{j}+\epsilon R_{j})K\left(i,j\right)S_{i}-\psi \left(R_{i},D_{i};R\right)S_{i},\\ \frac{dI_{i}}{dt} & =\sum_{j=0}^{15}\beta (I_{j}+\epsilon R_{j})K\left(i,j\right)S_{i}-\alpha I_{i}-\psi \left(R_{i},D_{i};R\right)I_{i},\\ \frac{dR_{i}}{dt} & =\alpha I_{i}-\phi \left(R\right)R_{i},\\ \text{where}\; & K\left(i,j\right)=e^{-\frac{d(i,j)}{r_{0}}}. \end{array} \end{array}$$ (1)

The parameter *β* is the transmission rate, *α* is the AI virus progression rate to infective farms, and *ϵ* is the reduction factor of the virus transmissibility for the reported farms. Susceptible farms in town *i*, *S*_*i*_, can be infected by two ways: either random contacts with infectious farms in the same town, *I*_*i*_ and *R*_*i*_, or nonrandom contacts with infectious farms from other towns, *I*_*j*_ and *R*_*j*_, for *j* = 0, …, 15 and *j* ≠ *i*. Here, contacts between domestic poultry farms represent indirect ways likely to transmit the AI virus via vehicles related to domestic poultry industry. Assuming density-dependent transmission, we use an exponential decay function with respect to the distance between two towns *i* and *j*, *d*_*ij*_, as a transmission kernel for nonrandom contact with infectious farms outside the local town *i*, $K\left(i,j\right)=e^{-\frac{d(i,j)}{r_{0}}}$, *r*_*o*_ is the scaling constant of the transmission kernel. The distance matrix whose the entry in the *i*-th row and *j*-th column is *d*_*ij*_ is estimated by the shortest path (road) in a map between two towns instead of the Euclidean distance between them. This estimation is reasonable in the case when neighboring two towns have no direct path between them because of some geographical reasons, such as rivers or mountains.

Culling is a localized control measure; it is only implemented in the premises affected by the AI virus. To add this spatial heterogeneity to the model we extend the PE culling rate function. We assume that the susceptible and infective poultry farms in town *i* are preemptively culled at a rate *ψ*(*R*_*i*_, *D*_*i*_; **R**) when there are AI-reported farms in town *i*. It is supposed that saturation of culling rate is dependent on the total number of reported farms across all towns, i.e., $R=\sum_{i=0}^{15}R_{i}$. Then, we let $\psi \left(R_{i},D_{i};R\right)=\frac{\eta D_{i}R_{i}}{A+R}$ with *A* > 0. The PE culling rate, *ψ*(*R*_*i*_, *D*_*i*_; **R**), linearly increases with respect to the number of reported cases in town *i* once the outbreak occurs, i.e., *ψ* ≈ *ηD*_*i*_*R*_*i*_, and the fraction of culling area in town *i*, *D*_*i*_. Finally, the PE culling rate does not continue to increase indefinitely when there exist reported farms in excess. Similarly, we assume that the IP culling is delayed by the total number of reported farms in all towns. Then, we let $\phi \left(R\right)=\frac{\gamma B}{B+R}$ with *B* > 0. Hence the IP culling rate decreases as the total number of reported cases across all towns increases.

We now introduce a metapopulation model with two-type poultry farms: chicken and duck. For *i* = 0, …, 15, *S*_*i*_, *I*_*i*_ and *R*_*i*_ are divided into two poultry farms such as *S*_*ci*_, *I*_*ci*_, *R*_*ci*_, *S*_*di*_, *I*_*di*_ and *R*_*di*_, respectively, where the subscript *c* is for chicken and *d* for duck. Then the model with two different farms can be written for *i* = 0…, 15 as the following nonlinear differential equations:

$$\begin{array}{c} \begin{array}{cc} & \frac{dS_{ci}}{dt}=-\sum_{j=0}^{15}(\beta_{cc}\left(I_{cj}+\epsilon R_{cj}\right)+\beta_{cd}\left(I_{dj}+\epsilon R_{dj}\right))K\left(i,j\right)S_{ci}-\psi \left(R_{i},D_{i};R\right)S_{ci},\\ & \frac{dI_{ci}}{dt}=\sum_{j=0}^{15}(\beta_{cc}\left(I_{cj}+\epsilon R_{cj}\right)+\beta_{cd}\left(I_{dj}+\epsilon R_{dj}\right))K\left(i,j\right)S_{ci}-\alpha_{c}I_{ci}-\psi \left(R_{i},D_{i};R\right)I_{ci},\\ & \frac{dR_{ci}}{dt}=\alpha_{c}I_{ci}-\phi \left(R\right)R_{ci},\\ & \frac{dS_{di}}{dt}=-\sum_{j=0}^{15}(\beta_{dc}\left(I_{cj}+\epsilon R_{cj}\right)+\beta_{dd}\left(I_{dj}+\epsilon R_{dj}\right))K\left(i,j\right)S_{di}-\psi \left(R_{i},D_{i};R\right)S_{di},\\ & \frac{dI_{di}}{dt}=\sum_{j=0}^{15}(\beta_{dc}\left(I_{cj}+\epsilon R_{cj}+\beta_{dd}\left(I_{dj}+\epsilon R_{dj}\right)\right))K\left(i,j\right)S_{di}-\alpha_{d}I_{di}-\psi \left(R_{i},D_{i};R\right)I_{di},\\ & \frac{dR_{ci}}{dt}=\alpha_{d}I_{di}-\phi \left(R\right)R_{di},\\ \text{where}\; & K\left(i,j\right)=e^{-\frac{d(i,j)}{r_{0}}}. \end{array} \end{array}$$ (2)

The parameter $\beta_{k_{1}k_{2}}$, for *k*_1_, *k*_2_ ∈ {*c*, *d*}, denotes the transmission rate from *k*_1_ farm to *k*_2_ farm. For example, *β*_*cd*_ represents the transmission rate from duck farms to chicken farms. For model simplicity, we suppose that interactions between chicken and duck farms are symmetric, i.e., *β*_*cd*_ = *β*_*dc*_. The parameters *α*_*c*_ and *α*_*d*_ denote the progression rate of chicken and duck farms, respectively.

The parameters *β*_*cc*_, *β*_*cd*_, *β*_*dd*_, *r*_0_, *η* and *B* were estimated using the least squares fitting method. The model prediction $C\left(t\right)=\int_{t_{0}}^{t}\psi \left(R\left(\tau \right)\right)I\left(\tau \right)+\phi \left(R\left(\tau \right)\right)R\left(\tau \right)d\tau$ was fitted to the cumulative number of AI-reported farms which is the summation of the cumulative number of PE culled farms that are confirmed as AI-positive and the cumulative number of IP culled farms. The built-in routine *lsqcurvefit* in MATLAB was used to solve the nonlinear least-squares problem.

As the first case was reported at a duck farm in Maengdong-myeon during the 2016-17 HPAI epidemic, we set *R*_*di*_(0) = 1 for *i* = 0 (indicating the infection source town), and *R*_*di*_(0) = 0 for *i* = 1, …, 15. For chicken, there were no initial cases, thereby setting *R*_*ci*_(0) = 0 for all *i*. Although it was found that there were a few farms already infected when the first case was reported [30], it might be hard to measure the exact number of those farms even by epidemiological surveillance. Therefore, the initial number of infective duck farm *I*_*d*_(0) in Maengdong-myeon was also estimated through the least-squares fitting method.

### Reproductive numbers

When an infected individual invades the susceptible population, the average number of secondary infection generated by the primary case over the infectious period, called the *basic reproductive number* and denoted by $R_{0}$, is an important threshold quantity [31–33]. In this work, to find the basic reproductive number, we use the next generation method [33, 34]. Let **G** be the next generation matrix, then $R_{0}=\rho \left(G\right)$ where *ρ* is the spectral radius. The (*i*, *j*) element of **G** means how many new infections are introduced into compartment *i* by the infected from compartment *j*. We now define the *local* reproductive number in town *j*, $R_{0}^{\left(j\right)}$, as how many poultry farms are newly infected by infected poultry farms from town *j*, and it is obtained by the maximum value among the farming types in each town after the sum of each column of **G**. As we consider two types of poultry farms, the next generation matrix can be written as a 2×2 block matrix,

$$G=\left[\begin{array}{cc} \begin{array}{c} G_{cc} \end{array} & \begin{array}{c} G_{cd} \end{array}\\ \begin{array}{c} G_{dc} \end{array} & \begin{array}{c} G_{dd} \end{array} \end{array}\right]$$ (3)

where the block $G_{k_{1}k_{2}}$ for *k*_1_, *k*_2_ ∈ {*c*, *d*} is a 16×16 matrix, and the entry of $G_{k_{1}k_{2}}$ is given by

$$\begin{array}{c} G_{k_{1}k_{2}}\left[i,j\right]=\beta_{k_{1}k_{2}}S_{k_{1}i}\left(0\right)(\frac{1}{\alpha_{k_{2}}}+\frac{\epsilon }{\gamma })K\left(i,j\right). \end{array}$$ (4)

A detailed report can be found in [33].

## Results

### Parameter estimations

In our study, the progression rate of chicken and duck farms, the initial IP culling rate, and the culling radius were set as *α*_*c*_ = 1/2, *α*_*d*_ = 1/4, *γ* = 1, and *r*_*c*_ = 3, respectively [1]. We assumed that saturation factor for the PE culling rate, which is the farm number where the culling rate reaches half of its maximum, *A* = 1 and the infectivity reduction factor, *ϵ* = 0.01. The model parameters are listed in Table 2.

**Table 2:** Model parameters.

Symbol	Description	Value	References
*β*_*cc*_	transmission rate between chicken farms	0.00441	data-fitted
*β*_*dd*_	transmission rate between duck farms	0.00707	data-fitted
*β*_*cd*_	transmission rate between different species	0.00007	data-fitted
*α*_*c*_	progression rate of chicken farms	1/2	[1]
*α*_*d*_	progression rate of duck farms	1/4	[1]
*r*_0_	scaling constant of the transmission kernel	4.5019	data-fitted
*γ*	initial infectious premises culling rate	1	[1]
*η*	PE culling rate	0.0618	data-fitted
*A*	decay constant for PE culling	1	assumed
*B*	decay constant for IP culling	2.995	data-fitted
*r*_*c*_	culling radius	3	[1]
*ϵ*	infectivity reduction factor	0.01	assumed

**Fig 3:**
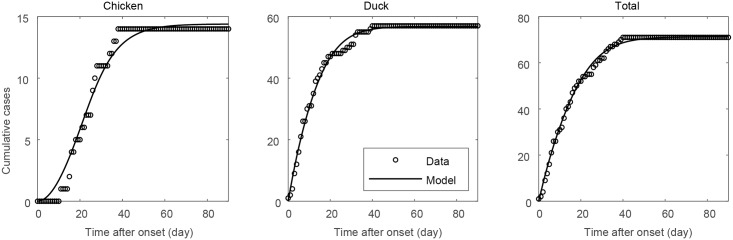
In each panel, circles represent the AI-reported farm data and black solid curves represent the corresponding simulation results.

Fig 3 displays the cumulative number of AI-positive farm (circles) and the best-fitted model results (solid curves) after the first case was reported on November 16, 2016, in Maengdong-myeon (Index 0). The fitted model agrees well with the observed data for both types of farms. Note that the transmission rate between duck farms (0.00707, 95% confidence interval: 0.00484-0.00930) is approximately 1.6 times higher than the transmission rate between chicken farms (0.00441, 95% confidence interval: 0.00183-0.00700). Interestingly, even though we only estimated non-spatial parameters such as transmission rates and scaling constants in the metapopulation model with two species, the total number of AI-positive farms from the model demonstrated a good fit to the corresponding data in most towns; the root-mean-square errors between data and model for chicken, and duck and total farms of the sixteen towns are 0.9392, 1.5020 and 1.5572, respectively.

### Reproductive numbers

**Fig 4:**
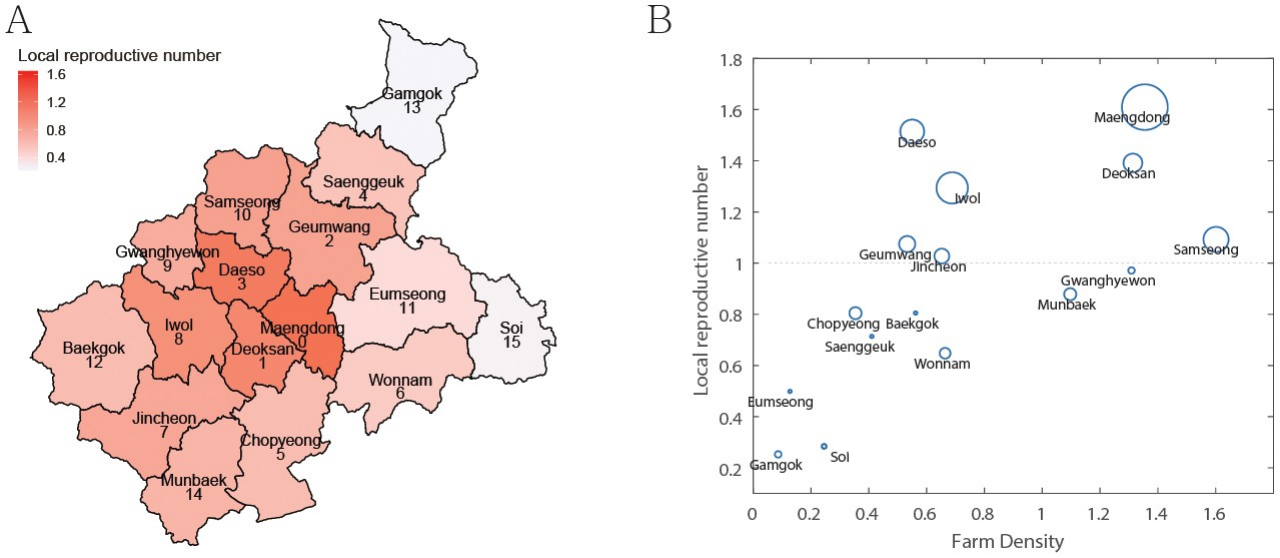
(A) Local reproductive numbers in each town. The darker red color represents the larger reproductive number. (B) Local reproductive number with respect to farm density in each town. The size of circle shows the size of duck farms. Reprinted from [27] under a CC BY license, with permission from National Geographic Information Institute, original copyright 2017.

Based on the initial population of poultry farms in Table 1 and the estimated parameters in Table 2, the estimate $R_{0}$ for the AI outbreak was 1.3427. Fig 4 displays the local reproductive number of each town by a map and graph which shows the correlation among local reproductive number, the farm density and the number of duck farms. In Fig 4, the towns with the local reproductive number greater than 1 are Maengdong ($R_{0}^{\left(0\right)}=1.6095$), Daeso ($R_{0}^{\left(3\right)}=1.5144$), Deoksan ($R_{0}^{\left(1\right)}=1.3913$), Iwol ($R_{0}^{\left(8\right)}=1.2940$), Samseong ($R_{0}^{\left(10\right)}=1.0926$), and Jincheon ($R_{0}^{\left(7\right)}=1.0277$). In most towns, the local reproductive numbers are positively correlated to the farm density (with correlation coefficient, *ρ* = 0.6606). Furthermore, the local reproductive numbers are also positively correlated to the number of duck farms (with correlation coefficient, *ρ* = 0.9383).

### PE culling strategy

**Fig 5:**
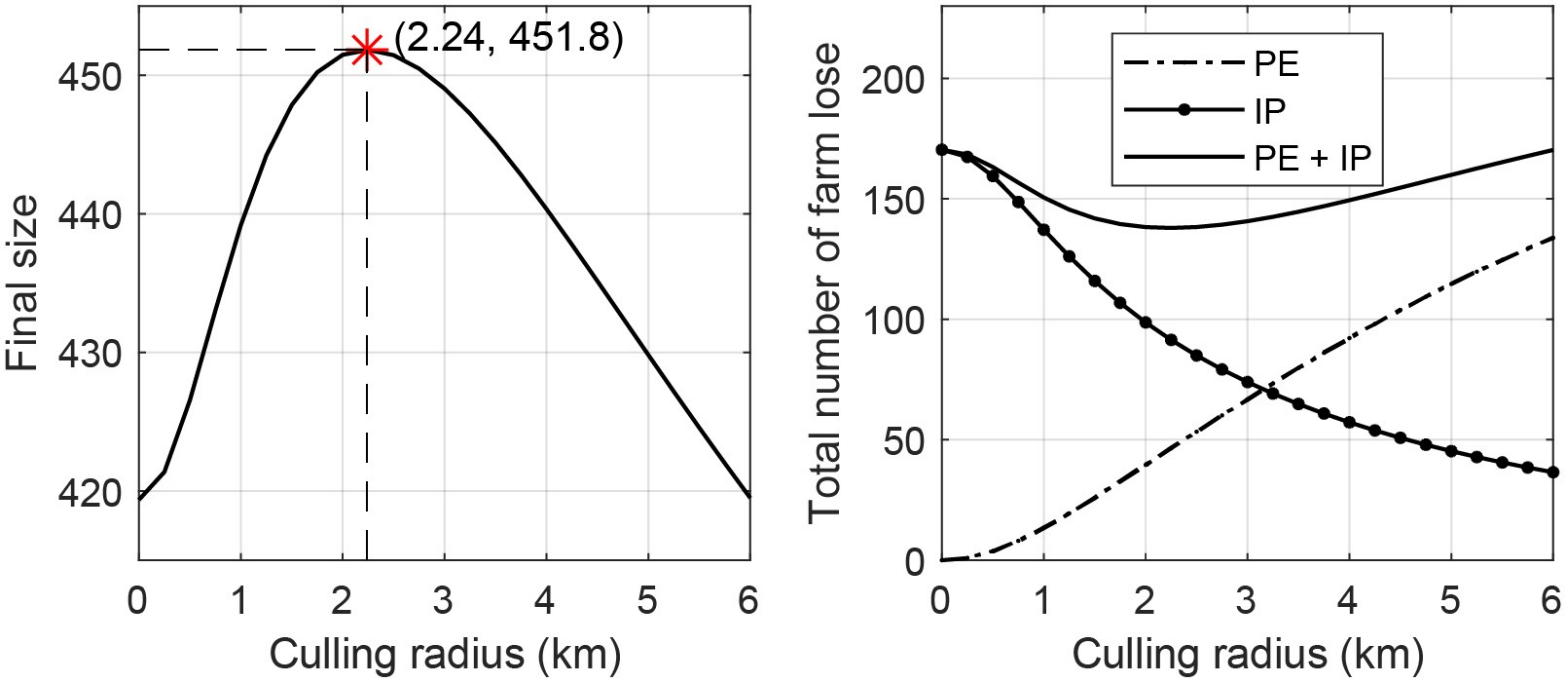
Impact of culling radius on the AI outbreak using the metapopulation model (Eq (2)) with all other parameters in Table 2. The left panel depicts the final size of susceptible farms with respect to culling radius. The right panel displays the total number of PE culling (dot-dashed), IP culling (dashed) and both (solid) with respect to culling radius. Note that when culling radius is 2.24 *km*, the final size of susceptible farms is maximized and the total number of culling is minimized.

Fig 5 shows the impact of culling radius on the AI outbreak using the metapopulation model (Eq (2)) with all other parameters in Table 2. The left panel depicts the final size of susceptible farms with respect to culling radius. The right panel displays the total number of PE culling (dot-dashed), IP culling (dashed) and both (solid) with respect to culling radius. A larger culling radius increases the loss by PE culling and suppresses outbreaks thereby decreases the loss by IP culling. This implies that the two culling strategies are in a compensatory relationship. The total loss of farms by culling reaches its minimum at *r*_*c best*_ = 2.24 *km* of culling radius in which the final size of the AI epidemic reaches its peak.

**Fig 6:**
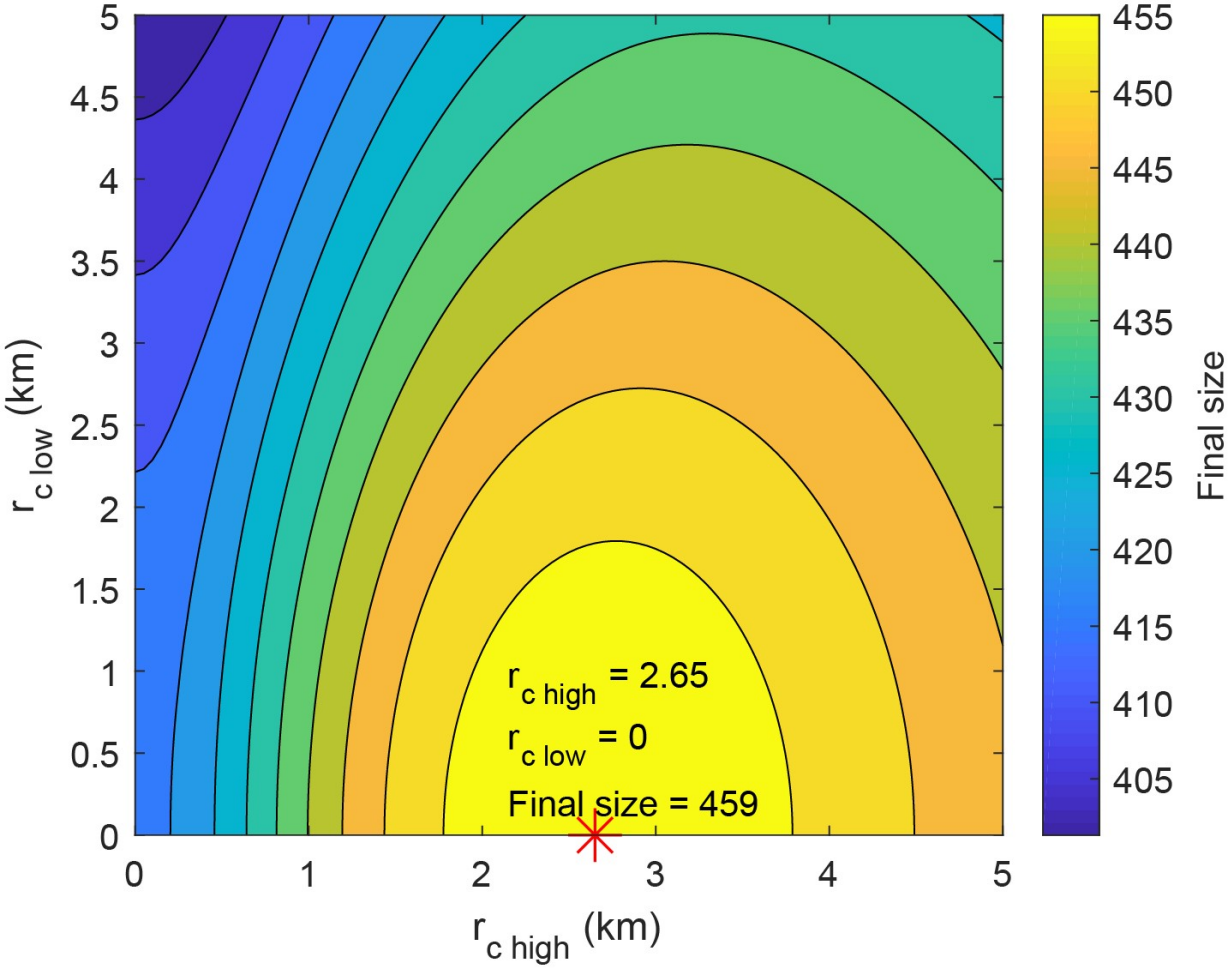
Impact of local-dependent culling radii on the final size of susceptible farms. The final size of susceptible farms with respect to culling radii is maximized as 459 when *r*_*c high*_ and *r*_*c low*_ are 2.65 and 0, respectively.

Although we found the best culling radius, *r*_*c best*_, at which the total loss of farms by culling reaches its minimum, spatial heterogeneity was not considered. We set culling radius as localized parameter; *r*_*c high*_, culling radius of towns with $R_{0}^{\left(i\right)}>1$ and *r*_*c low*_, culling radius of towns with $R_{0}^{\left(i\right)}<1$. Fig 6 depicts the impact of the two localized culling radii on the final size of susceptible farms by a color bar. In the parameter domain, the final size of susceptible farms along the axis *r*_*c high*_ dramatically increases at first, then slowly decreases later. Meanwhile, the final size of susceptible farms decreases as *r*_*c low*_ increases. The final size of susceptible farms is maximized as 459 when *r*_*c high*_ and *r*_*c low*_ are 2.65 and 0, respectively. The best culling radius in towns with $R_{0}^{\left(i\right)}>1$, *r*_*c high*_ = 2.65 *km*, is smaller than the government’s policy (3 *km*), but greater than one ignoring the spatial heterogeneity, *r*_*c best*_ = 2.24 *km*.

## Discussion

Using farm-to-farm transmission dynamics incorporating the two poultry types, chicken and duck, we estimated the spread of the HPAI outbreak in the Republic of Korea in 2016-2017. We found that from modeling result the transmissibility between duck farms was higher than that between either chicken farms or different type of farms. Ducks can carry and shed the AI virus without symptoms and have low mortality while chickens have high pathogenicity and mortality [5]. In addition, the outbreak started at a duck farm in high farm density area. These epidemiological and spatial factors amplified the virus and allowed it to spread easily surrounding poultry farms. Therefore, duck farms played an important role in the spread of the HPAI virus in Eumseong-gun and Jincheon-gun. This is the first study on estimating the basic reproductive number of the 2016-2017 HPAI epidemic in the Republic of Korea and introducing the local reproductive number as an indicator of the virus transmission in a given area. There were six towns in which the local reproductive number is greater than 1: Maengdong ($R_{0}^{\left(0\right)}=1.6095$), Daeso ($R_{0}^{\left(3\right)}=1.5144$), Deoksan ($R_{0}^{\left(1\right)}=1.3913$), Iwol ($R_{0}^{\left(8\right)}=1.2940$), Samseong ($R_{0}^{\left(10\right)}=1.0926$), and Jincheon ($R_{0}^{\left(7\right)}=1.0277$). These towns seem to be either relatively close to Maengdong (Deoksan, Geumwang and Daeso) or have high farm density (Maengdong, Deoksan and Samseong). The distance from Maengdong to Deoksan, Geumwang and Daeso are 7.76 *km*, 9.77 *km* and 9.95 *km*, respectively, which are almost half of mean distance (18.31 *km*) to Maengdong. Since the transmission rate depends on the distance between towns by the kernel, $K\left(i,j\right)=e^{-\frac{d(i,j)}{r_{0}}}$, it is clear that the local reproductive number is affected by the distance. The high farm density in Deoksan and Samseong (1.31 and 1.60, respectively) might allow the virus to spread easily surrounding poultry farms via movement of humans (farm personnel and visitors) and vectors (rodents), or contaminated environment (air and water) [35].

Maengdong, which is the town with the highest local reproductive number, is the region with the highest number of duck farms and the source of outbreak. The three towns with the lowest local reproductive number are Gamgok, Soi, and Eumseong, which also have the lowest farm density. These imply that farm density plays an important role on the 2016-2017 HPAI epidemic in the Republic of Korea, which is in line with the previous works on livestock diseases [19, 20, 36–40]. Furthermore, we found that $R_{0}^{\left(i\right)}$ is strongly influenced by not only epidemiological factors, such as the number of duck farms with high transmissibility in town *i*, but also by geographical factors, such as the location of town *i* and its proximity to other towns with high susceptibility.

Our finding showed that culling strategies need to take heterogeneity into account for reducing loss of poultry farms. We suggested that PE culling has to be focused on the area in which the local reproductive number is greater than 1, and the culling radius must be greater than that, ignoring the spatial heterogeneity; Reinforced PE culling in the area wherein the local reproductive number is greater than 1 could allow for early depopulation of infected farms before infection spreads to dense area. Meanwhile, in the areas in which the local reproductive number is less than 1, losses from the infection might be greater than losses from PE culling.

## Conclusions

In this paper, we presented mathematical modeling for the spatial-temporal transmission dynamics of HPAI using geographical and epidemiological data of the 2016-2017 AI epidemic in the Republic of Korea. To consider spatial heterogeneity, we introduced the PE culling rate as a function of the number of reported farms [15] with different coefficients based on the farm density in each town. As the exact location of the poultry farms affected by the AI virus was not available, a metapopulation framework has been adopted with the two types of poultry farms assuming random mixing between farms in a patch and nonrandom contact by the transmission kernel [19, 41] between farms in different patches. We introduced the local reproductive numbers using the next generation method [33, 34] in the metapopulation modeling framework. This quantified parameter allowed one to assess the transmissibility in each town. For total and even both types of farms, the model predictions and data about the AI-reported farms are in good agreements. The estimated transmission rates showed that the transmission between duck farms played an important role in the spread of the HPAI virus in Eumseong-gun and Jincheon-gun.

Our findings showed that the local reproductive number could be an indicator of the likelihood of virus transmission in a given area. It was revealed that this parameter was strongly influenced not only by epidemiological factors such as transmissibility and/or susceptibility of poultry species but also by geographical and demographical factors such as the distributions of poultry farms (or density) and connectivity (or distance) between farms. Based on this result, we found that the culling radius of PE culling should be adjusted by considering the local reproductive number in the target area. Therefore, to determine which area is supposed to be more strictly controlled during an AI outbreak, we believe that veterinary/public health officials can use the local reproductive number in a real-time warning system. Our study can be applied to other animal diseases (e.g., foot-and-mouth disease [42], brucellosis [25]) in which such heterogeneities are crucial factors to be considered.

## Supporting information

S1 Table Time series outbreak data (chicken farms) of the sixteen towns [1]. (CSV)

S2 Table Time series outbreak data (duck farms) of the sixteen towns [1]. (CSV)

S3 Table Pairwise distance matrix of the sixteen towns (*km*). (CSV)

S4 Table Daily AI report and IP culling number. This are gathered and translated from HPAI infection daily reports of the Ministry of Agriculture, Food and Rural Affairs [1].
(CSV)
